# Opioid-free anesthesia: the next frontier in surgical patient safety

**DOI:** 10.1186/s13037-022-00346-5

**Published:** 2022-12-02

**Authors:** Jason McLott, Philip F. Stahel

**Affiliations:** 1 Mission Health, 50 Schenck Pkwy, Asheville, NC 28803 USA; 2 Department of Surgery, East Carolina University, Brody School of Medicine, E. 5th St., Greenville, NC 27858 USA; 3 Department of Specialty Medicine, Rocky Vista University, College of Osteopathic Medicine, 8401 S. Chambers Rd., Parker, CO 80134 USA

**Keywords:** Opioid epidemic, Enhanced surgical recovery, Opioid-free anesthesia, Patient safety

The nationwide opioid addiction epidemic was in large part boosted by the surgeons’ liberal practice of prescribing opioids for perioperative pain control in the 1990s [1]. In the United States alone, an estimated 500,000 people died between 1999–2019 from opioid overdose [2]. Historically, opium has been used as part of sleep-inducing sponges (*“spongia somnifera”*) for pain control during surgical procedures as far back as in the medieval times [3]. Prior to the introduction of anesthesia for surgical procedures, it was not uncommon for patients to succumb to the shock induced by surgical pain. With the discovery of volatile anesthetics in the nineteenth century, surgeons were finally able to perform procedures on patients who were unresponsive to the surgical pain [4]. At present, there are two main techniques for delivering general anesthesia: volatile/inhaled anesthesia and total intravenous anesthesia [5]. Opioids were introduced to the anesthesia practice as a method of decreasing the amount of volatile anesthetic necessary for surgical anesthesia, thereby decreasing the risk of toxic side effects from volatile agents [4]. Opioids work synergistically with volatile anesthetics and also provide essential analgesic benefits [6]. The morphine-like opioids used in general anesthesia provide analgesia by binding to *mu* (μ) receptors [7]. *Mu* receptor agonism is associated with significant perioperative side effects, including respiratory depression, postoperative nausea/vomiting, constipation, and altered immunomodulatory signaling pathways [7]. Traditionally, the unfavorable side effect profile of morphine-like opioids has been tolerated as a necessary “collateral damage” of general anesthesia, and the management of these side effects has represented a routine part of perioperative care [8]. These traditional standards of opioid-based pain control during surgery and postoperative care have only recently been challenged in response to the recognition of the “iatrogenic” root cause of the widespread opioid addiction epidemic in the twenty-first century [2]. The “Enhanced Surgical Recovery” (ESR) protocol represents a modern streamlined approach designed to optimize the patients’ surgical care by reducing the use of opioids during the perioperative phase in favor of multimodal perioperative pain management protocols [9].

The intuitive next frontier for streamlining our patients’ perioperative care and improving surgical patient safety and patient outcomes is represented by the proactive (and arguably provocative) concept of opioid-free anesthesia. The primary goal of opioid-free anesthesia is to abstain from the use of *mu* receptor agonists through the use of non-steroidal anti-inflammatory drugs (NSAIDs), acetaminophen, lidocaine, dexmedetomidine, ketamine, and low-dose glucocorticoids. In addition, regional nerve blocks represent a fundamental pillar of intra- and postoperative analgesia as part of the opioid-free anesthesia protocol [10]. Dexmedetomidine is a selective α2-adrenergic receptor agonist with sedative and analgesic properties. The concept of dexmedetomidine-based anesthesia leverages the benefits of the multimodal pain control strategy in the perioperative arena to decrease both peripheral and central sensitization [11]. Notably, this new modality of general anesthesia has been shown to significantly reduce opioid consumption in patients undergoing bariatric surgery [12], pancreatic surgery [13], major urological procedures [14], rotator cuff repair [15], and total hip replacement [16]. A recent randomized controlled trial in gynecological surgery furthermore corroborated that opioid-free anesthesia was associated with significant improvement in postoperative analgesia, compared to traditional opioid-based anesthesia [17]. At our own institution, opioid-free anesthesia represents the modality of choice for reducing the risks associated with traditional opioid-based anesthesia.

Table 1 outlines the respective medications and dosage ranges for opioid-based vs. opioid-free anesthesia. The first author has a 6-year personal experience with opioid-free anesthesia and delivered the proof-of-concept that this proactive concept is indeed feasible, safe, and effective in providing general anesthesia for all surgical procedures (Jason McLott, unpublished observations). Considering the fact that more than 200 million surgeries are performed world-wide every year [18], the new proactive concept of opioid-free anesthesia likely represents the next frontier for surgical patient safety on a global scale.

**Table 1:** Medications and dosages in opioid-based *vs.* opioid-free anesthesia

Opioid-based	Opioid-free
	*Induction (boluses)*
Propofol 1.5–2 mg/kg	Propofol 1 mg/kg
Fentanyl 1–5 μg/kg	Dexmedetomidine 0.2–0.5 μg/kg (incremental dosages)
Versed 1–5 mg	Ketamine 0.25–0.5 mg/kg
Lidocaine 1.5 mg/kg	Lidocaine 2 mg/kg
	Toradol 15–30 mg
	*Maintenance Infusion (“McLott Mix”)*
	Dexmedetomidine 0.4 μg/kg/hr
	Ketamine 0.3 mg/kg/hr
	Lidocaine 2 mg /kg/hr
	Magnesium 10 mg /kg/hr

## Data Availability

Please contact the authors for data requests.
